# Functional Changes in the Fallopian Tube: Environmental Factors, Lifestyle, Pathological Conditions and Pharmacological Agents

**DOI:** 10.3390/cells15030269

**Published:** 2026-01-31

**Authors:** Opalina Roy, Sandhya Kumari, Satish Kumar Adiga, Manjunath B. Joshi, Anujith Kumar, Ganesh Venkatraman, Nagarajan Kannan, Guruprasad Kalthur

**Affiliations:** 1 Division of Reproductive Biology, Department of Reproductive Science, Kasturba Medical College, Manipal, Manipal Academy of Higher Education, Manipal 576104, Karnataka, India; opalina.roy@learner.manipal.edu (O.R.); sandhya.patil@manipal.edu (S.K.); 2 Centre of Excellence in Clinical Embryology, Department of Reproductive Science, Kasturba Medical College, Manipal, Manipal Academy of Higher Education, Manipal 576104, Karnataka, India; 3 Department of Ageing Research, Manipal School of Life Sciences, Manipal Academy of Higher Education, Manipal 576104, Karnataka, India; manjunath.joshi@manipal.edu; 4 Manipal Institute of Regenerative Medicine, Bangalore 560065, Karnataka, India; 5 School of Biosciences and Technology, Vellore Institute of Technology, Vellore 632014, Tamil Nadu, India; 6 Division of Experimental Pathology and Laboratory Medicine, Department of Laboratory Medicine and Pathology, Mayo Clinic, Rochester, MN 55902, USA; kannan.nagarajan@mayo.edu; 7 Center for Regenerative Medicine, Mayo Clinic, Rochester, MN 55902, USA; 8 Mayo Clinic Cancer Center, Mayo Clinic, Rochester, MN 55902, USA

**Keywords:** endocrine disruptors, epithelial multilayering, sperm transport, ectopic pregnancy, infertility

## Abstract

The fallopian tubes are critical segments of the female reproductive tract and are essential for transporting gametes and embryos. It creates a conducive environment necessary for successful fertilization, early embryo development, and embryo transport. The cellular composition and function of the fallopian tube are tightly regulated by the sex hormones estradiol and progesterone. Therefore, any pathological/ metabolic condition or exposure to exogenous agents with the potential to alter endocrine levels can have a significant impact on fallopian tube function and health. This review summarizes the effects of medications, infections, pathological conditions, lifestyle choices, and environmental factors that can significantly impact the morphology, histology, cellularity, and functionality of the fallopian tube.

## 1. Introduction

The fallopian tubes are crucial parts of the female reproductive system, responsible for the transport of sperm, oocytes and embryos. In addition, the fallopian tubes temporarily store partially capacitated spermatozoa for up to 3 days and play a crucial role in creating a favorable environment for successful fertilization and pre-compaction stage embryo development [1]. The tube has unique immune features [2] and is where interesting events such as chemotaxis and thermotaxis occur to facilitate sperm–oocyte approximation during fertilization. Further it orchestrates the movement of spermatozoa towards the infundibular end, while oocyte and embryo are pushed in the opposite direction, towards the uterine ostium.

The fallopian tube is a tubular, seromuscular organ connected distally to the ovary and proximally to the lateral aspect of the uterine fundus, supported along its length by the mesosalpinx. Morphologically and anatomically, the fallopian tube can be divided into five segments: (1) the infundibulum, which terminates in the fimbriated end surrounding the abdominal ostium of the tube; (2) the ampullary region; (3) the isthmic portion; (4) the intramural or interstitial portion, located within the uterine wall; and (5) a small uterotubal junction (UTJ) [3]. Fimbriae aids in the oocyte-cumulus complex pick-up process following follicle rupture. Fertilization process occurs in ampulla and the developing embryo travels through the isthmus and reaches uterine cavity via the uterotubal junction.

**Figure 1:**
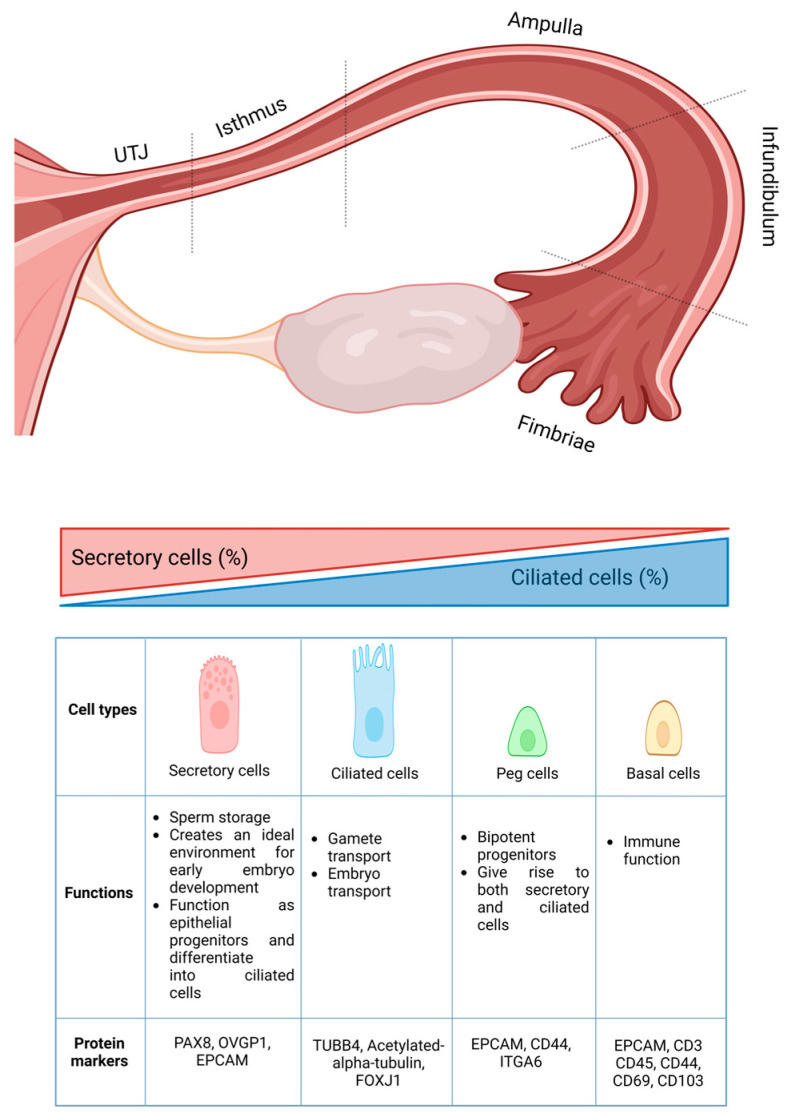
Schematic representation of the fallopian tube anatomy, highlighting its cellular composition and key characteristics of the constituent cell types (PAX8: Paired-box gene 8; OVGP1: Oviductal glycoprotein 1; EPCAM: Epithelial cell adhesion molecule; TUBB4: Tubulin beta 4; FOXJ1: Forkhead box J1; ITGA6: Integrin subunit alpha). Created with BioRender.com.

The fallopian tube is lined with a single layer of cuboidal or columnar mucosal epithelium. Traditionally, the epithelial lining the fallopian tube is composed of four types of cells: (1) non-ciliated secretory cells, which make up about three-fourths of the total population; (2) ciliated cells, which constitute around one-fourth; a minor population of (3) peg cells; and (4) basal cells (Figure 1) [4,5,6]. The distribution of the secretory and ciliated epithelial cells varies along the entire fallopian tube, with a higher proportion of multiciliated cells found in the infundibulum (over 50% in the fimbria) in contrast to a lower proportion observed in the isthmus and utero-tubal junction (less than 35%) [5,7]. The secretory cells play crucial roles in sperm storage and create an ideal atmosphere for early embryo development, while the ciliated epithelial cells help in gamete and embryo transport [5]. Further, secretory cells under appropriate conditions function as epithelial progenitors and can differentiate into ciliated cells [6,7]. The biological functions of peg and basal cells remain poorly understood [8]. However, few in vitro studies have demonstrated that peg cells can expand and form organoids containing both ciliated and secretory cells, indicating their potential stem cell or progenitor properties [9,10]. Histologically, basal cells are characterized by their round shape, a cytoplasmic ring, and the expression of epithelial (EPCAM) and T cell or resident T cell markers, suggesting a possible role in immune function [11,12].

Fallopian tubes are intricately regulated by dynamic changes in the endocrine level, which not only affects the contractility of the myosalpinx (smooth muscle layer of the tubal wall) but also modulates the viscosity of tubular fluids [13]. Fallopian tubes can sense the cyclical hormonal changes occurring in the ovary and can organize the tubal microenvironment accordingly since the arterial blood supply of the fallopian tube comes from branches of the uterine and ovarian arteries. Typically, branches of the uterine artery supply the isthmus and proximal ampulla, while branches of the ovarian artery supply the rest of the fallopian tube [3,14]. Therefore, changes in the fallopian tube occur much earlier than in the uterus during the menstrual cycle, which is probably customized to ensure the oocyte/embryo transport before preparing a receptive endometrium [14].

Hormones play a crucial role in the development and physiological function of the fallopian tubes. Ovarian steroidal hormones, especially estradiol (E2) and progesterone (P4), appear to drive cyclic changes in the ratio of ciliated cells to secretory cells in the fallopian tube [15,16]. Chen et al. [17] using porcine models have demonstrated that during the estrus stage, the proportion of ciliated cells is the highest, while in the diestrus stage, there is a high proportion of secretory cells. E2 promotes ciliation and accelerates the ciliary beat frequency (CBF) of ciliated cells in the fallopian tube, while P4 reduces ciliation and decreases CBF [1,18,19,20]. E2 enhances the contraction of tubal smooth muscle activity, leading to an increased rate of contractions, which in turn helps with sperm and oocyte transport. In addition, E2 levels rise prior to ovulation, mucin secretion by the secretory epithelial cells increases, causing the tubal luminal fluid to become less viscous, which helps in oocyte and sperm migration to the ampulla, the site of fertilization. In contrast, P4 reduces muscle contractility in the fallopian tube, promoting relaxation and increases the tubal fluid viscosity by reducing the mucin secretion [16,21].

**Figure 2:**
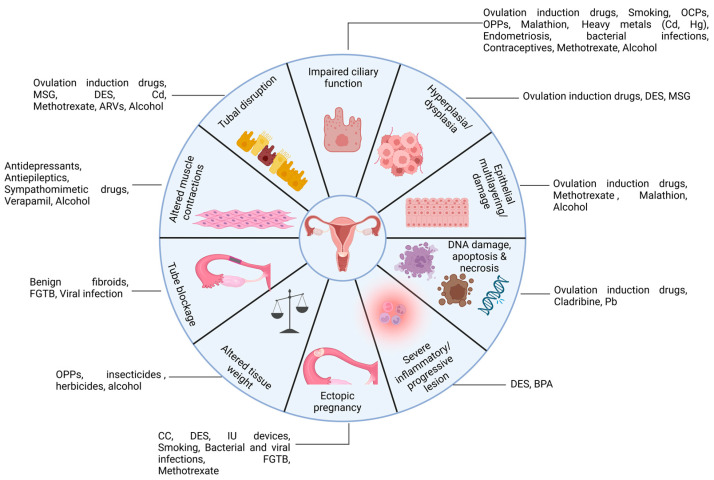
Schematic representation of the potential risk factors influencing structure and function of the fallopian tube. Created with BioRender.com.

Alterations in the sex hormone profile due to exogenous (environmental factors, lifestyle exposures) or endogenous (pathological conditions, medications) can disrupt fallopian tube morphology and function. Cytological changes such as epithelial dysplasia, hyperplasia, multilayering, tubal dilation, edema, necrosis, irregular folds, mucosal atrophy, nuclear atypia, blebbing, and the loss of ciliated cells and impaired smooth muscle contractility have been reported in experimental models with endocrine disruption (Table 1, Table 2 and Table 3). These changes can result in altered gamete and embryo transport and thereby increasing the risk of ectopic pregnancy and infertility. Given the growing threats to women’s reproductive health, addressing these multifactorial risks warrants urgent attention. This review provides a timely and accurate integration of current evidence on potential factors that contribute to the alterations in the structure and function of the fallopian tube (Figure 2), providing insights into their implications for fertility and reproductive health

## 2. Search Strategy and Selection Criteria

This review aims to enhance our understanding of how medications, infections, pathological conditions, lifestyle factors, and environmental exposures influence the morphology, histology, cellular composition, and functional integrity of the fallopian tube by critically analyzing and summarizing existing evidence. A comprehensive literature search was conducted across PubMed, Scopus, and Google Scholar, yielding 725 records, with no start date applied. The search was performed manually, without automated tools, using a combination of keywords and Medical Subject Headings (MeSH) terms, including: “fallopian tube”, “fallopian tube epithelial cells”, “pesticides, fallopian tube”, “insecticides, fallopian tube”, “herbicides, fallopian tube”, “endocrine disrupting chemicals, fallopian tube”, “heavy metals, fallopian tube”, “food additives, beverages, fallopian tube”, “smoking, fallopian tube”, “alcohol, fallopian tube”, “electromagnetic radiation, fallopian tube”, “infections, fallopian tube”, “medical conditions, fallopian tube”, “pharmacological agents, fallopian tube”, “anticancer and antiviral drugs, fallopian tube”, “contraceptives, fallopian tube”, and “ovulation induction drugs, fallopian tube”.

After removing duplicates, 580 records were screened, of which 284 full-text articles were retrieved for detailed evaluation. The remainder were excluded as irrelevant or lacking essential information. Reference lists of relevant articles and reviews were also screened manually for additional studies. Ultimately, 191 articles were assessed for eligibility, and the search was restricted to English language publications. Inclusion criteria comprised original research articles, clinical trials, narrative reviews, systemic reviews and meta-analyses reporting the impact of medications, infections, pathological conditions, lifestyle factors, or environmental exposures on fallopian tube structure and function. Exclusion criteria included abstracts, conference proceedings, opinion pieces, anecdotal evidence, articles under review, and non-English publications. In total, 187 articles met the eligibility criteria, spanning from June 1968 (earliest relevant article) to July 2024 (most recent relevant report).

The review first outlines the structure, function, and hormonal regulation of the fallopian tube, then discusses in detail how diverse threats compromise its integrity and human reproductive health and concludes that safeguarding women’s reproductive wellbeing requires minimizing environmental and lifestyle risks while exercising caution with pharmacological interventions. As this is a narrative review, a formal risk of bias assessment was not performed. However, the strengths and limitations of the cited studies were considered, including study design (in vitro, in vivo, or clinical), sample size, methodological variability, and relevance to human physiology. Potential sources of bias such as publication bias, selective reporting, and heterogeneity across experimental models are acknowledged. These limitations may affect the generalizability of the findings, and conclusions should therefore be interpreted with caution, taking into account the absence of standardized evidence grading system when considering the overall synthesis.

## 3. The Environmental Toxicants

The female reproductive system is highly sensitive to environmental influences, which can disrupt its normal physiological functions. Exposure to various environmental pollutants has been increasingly linked to reproductive health disorders, including infertility, hormonal imbalances, and cancers of reproductive organs [22,23,24,25]. Even though a variety of environmental toxicants can potentially alter the structure and function of fallopian tube, pesticides, insecticides, heavy metals, food additives, and agents with endocrine disrupting functions are reported to have detrimental effects on the physiology and function of the fallopian tube (Table 1).

**Table 1:** Impact of environmental toxicants and lifestyle choices on fallopian tube function.

Compound/Factor	Changes Induced in the Fallopian Tube	Organism	Reference
Organochlorine pesticides (DDT, MXC, TCMP)	Impaired ciliary movement	Hamster, Bovine, Alligator	Tiemann et al., 1999 [26]; Pöhland et al., 2003 [27]; Yan et al., 2011 [28]; Vonier et al., 1996 [29]; Sharma et al., 2016 [30]
Organophosphate pesticides (TZ, MP)	Increased oviduct weight, loss of microvilli and significant reduction in kinocilia	Rat	Guney et al., 2007 [31]; Bhanot et al., 2020 [32]
Acetamiprid	Reduced oviduct length, damaged lumen	Silkworm	Cheng et al., 2019 [33]
Methomyl, Cypermethrin, Paraquat	Reduced oviduct weight	Mouse, Hen	Shanthalatha et al., 2012 [34]; Nada et al., 2017 [35]; He et al., 2021 [36]
Malathion	Disruption of mucosal lining of the isthmus, damage to both secretory and ciliated cells	Goat	Sharma et al., 2016 [30]
Diethylstilbestrol (DES)	Tubal disruption, hyperplasia, DAO, salpingitis	Mouse	Newbold et al., 2004 [22]; Newbold et al., 1983 [37]; Newbold et al., 1995 [38]
	Severe inflammatory lesions	Hamster	Alwis et al., 2011 [39]
Bisphenol A (BPA)	Irregular mucosal folds	Mouse	Newbold et al., 2009 [40]; Sakali et al., 2024 [41]
Polychlorinated biphenyls (PCBs)	Impaired motor functions	Bovine	Wrobel et al., 2020 [42]
Cadmium	Blebbing and loss of ciliary cells	Hamster	Bhardwaj et al., 2021 [43]; Magers et al., 1995 [44]
	Tubal disruption, edema, fragmentation of nuclear chromatin	Rabbit	Massányi et al., 2020 [45]
	Hyperemia, extensive hemorrhage, cellular damage	Koel	Sarkar et al., 1976 [46]
Mercury	Damage to ciliated cells	Duck	Balachandran et al., 1985 [47]
Lead	Necrosis	Rat	Dumitrescu et al., 2015 [48]
Monosodium glutamate (MSG)	Tubal disruption, cellular hypertrophy	Rat	Eweka et al., 2010 [49]
Smoking (nicotine)	Impaired tubal motility	Monkey	Neri and Marcus, 1972 [50]
	Damage to ciliated cells, ciliary movement inhibited	Hamster	Magers et al., 1995 [44]; Knoll and Talbot, 1998 [51]
	Decreased blood flow	Rat	Phipps et al., 1987 [52]
Electromagnetic field (EMF)	Increased height of epithelial cells	Mouse	Rajaei et al., 2010 [53]

### 3.1. Pesticides, Insecticides, and Herbicides

Approximately 2.3 billion people, representing 32% of the global population, consume pesticides beyond the acceptable intake levels [54], and an estimated 3 million new cases of pesticide poisoning are reported worldwide annually, with over 10% resulting in death [55]. Even though not many studies in the literature indicate the consequences of pesticide exposure on human fallopian tube structure and function, indirect evidence suggests that pesticides have adverse effects on the fallopian tube function. Fuortes et al. [56] reported that those employed in agricultural-related industries had a 4 to 16 times greater risk of developing fallopian tube disorders along with a significantly increased risk of infertility compared to those involved in other occupations. Lerro et al. [57], in a prospective study conducted in Iowa and North Carolina, USA, as part of the Agricultural Health Study (AHS) involving pesticide applicators and their spouses, reported an increased incidence of ovarian and fallopian tube tumors among the spouses of agricultural workers. The analysis, based on 12,420 cancer cases accumulated over a twenty-year follow-up period, attributed the increased cancer risk to exposure to farming-related hazards including pesticides, diesel exhaust, ultraviolet radiation, biologically active dust, and zoonotic infections originating from livestock.

### 3.2. Endocrine-Disrupting Chemicals

Endocrine-disrupting chemicals (EDCs) are substances that interfere with the normal function of the endocrine system [23]. The impact of EDCs on human health is significantly more severe in low- and middle-income countries, where elevated human exposure has been associated with over 400,000 cases of low birthweight babies in the past two decades [58,59]. Gestational exposure to DES in humans caused both functional and anatomical abnormalities in the fallopian tubes of female offspring, highlighting potential transplacental effects [60].

## 4. Lifestyle

With civilization, a dramatic change in human lifestyle has been progressively observed. Global lifestyle changes, such as increased smoking, alcohol consumption, and exposure to electromagnetic radiation have shown to adversely affect human reproductive health including the physiology and function of human fallopian tube [61,62].

### 4.1. Caffeine

Over the past few decades, food additives have become a foundation of the modern food industry, driven by consumer preferences, and play a crucial role in enhancing the color, aroma, and palatability of food. Coffee is among the most widely consumed beverages globally. Caffeine (1,3,7-trimethylxanthine), a natural alkaloid found in coffee beans, tea leaves, kola nuts, and other plants, is also present in various other beverages (such as soft drinks and energy drinks), foods (including cocoa, chocolate, and guarana), sports supplements, and even medications [63]. Caffeine can reduce muscle activity in the fallopian tube, which may lead to decreased pregnancy rates in women [64].

### 4.2. Smoking

A global meta-analysis revealed that the overall prevalence of lifetime and current cigarette smoking among women was 28% and 17%, respectively. When stratified by subgroup, the prevalence of lifetime smoking was 23% among adolescent girls/students, 27% in adult women, 32% in pregnant women, and 38% in women with any disease [65]. Cigarette smoking disrupts fallopian tube epithelial turnover by downregulating BCL2-associated agonist of cell death (BAD) and upregulating BCL2, changes that might represent potential mechanisms contributing to structural and functional alterations [66]. Meanwhile, epidemiological data demonstrates a significantly increased risk of ectopic pregnancy among current smokers, with risk normalization observed within 10 years of cessation [67].

### 4.3. Alcohol

Alcohol consumption is prevalent across societies worldwide as a social practice or lifestyle. Globally, around one-third of the population (2.4 billion individuals) consume alcohol, with alcohol-related health problems accounting for 2.2% of deaths in women and 6.8% in men annually [68]. Several studies have demonstrated strong association between alcohol intake and reduced female fertility [69,70,71,72]. Ethanol impairs ovarian function [72] and disrupts fallopian tube transport by reducing basal tone as well as the amplitude and frequency of muscle contractions via the NO pathway [71].

## 5. Infections

Various bacterial and viral infections pose risks to female reproductive health by compromising the structural and functional integrity of the fallopian tubes [6,73,74,75,76,77,78]. Infections due to miscarriages, medical pregnancy terminations, puerperal infections, and the use of intrauterine contraceptive devices have shown to cause damage to the fallopian tubes [79]. Many studies have presented evidence suggesting that the fallopian tube may not have a sterile microenvironment [80,81,82]. Bacterial infections (*Chlamydia trachomatis* and *Neisseria gonorrhoeae*) significantly reduce CBF, ciliated cell proportion, and muscle contraction [73,74,75], increasing the risk of ectopic pregnancy [83]. Studies on women of reproductive age reported that the prevalence of *Chlamydia trachomatis*, *Mycoplasma genitalium*, cytomegalovirus (CMV), and herpes simplex virus (HSV)-1/2 was significantly higher in the fallopian tubes of women with ectopic pregnancy [77,84,85]. Further, HSV-2 has been identified in the fallopian tubes of women with acute pelvic inflammatory disease (PID) [77,86]. Viral and genital herpes infections have been demonstrated to increase inflammatory cells in the upper genital tract and activation of Toll-Like Receptors (TLRs) in fallopian tube epithelial cells that can elevate the risk of fallopian tube damage [77,78].

Female genital tuberculosis (FGTB) is a chronic infection of the female reproductive system caused by *Mycobacterium tuberculosis*. It is a major cause of tubal obstruction and infertility, particularly in women of underdeveloped and developing countries [87]. Over 90% of patients with FGTB experience fallopian tube blockage, calcification, hydrosalpinx, and adhesions [88]. Tubal occlusion most frequently occurs at the junction between the isthmus and ampulla, which can lead to the accumulation of serous or clear fluid, resulting in a sausage-shaped dilation of the tube, initially presenting as pyosalpinx and later transforming into hydrosalpinx. The dilation of the fallopian tube can lead to a “golf club appearance” [89,90,91], twisting may result in a “floral hydrosalpinx” pattern, intraluminal scarring can cause a cobblestone pattern [89,90,91], multiple strictures can create a beaded appearance, and severe scarring may produce a rigid, stem-like appearance [89,90,91,92]. Peritoneal adhesions are indicated by a convoluted or corkscrew-shaped tube and severe adhesions may lead to irregular septation and a “criss-cross spill pattern” on imaging [90,91]. Women with FGTB have low conception rates and face a significantly higher risk of ectopic pregnancy [93].

## 6. Medical Conditions

Medical conditions such as hydrosalpinx, salpingitis, endometriosis, benign fibroids, PID, and prior abdominal or pelvic surgeries, including surgery for ectopic pregnancies (Table 2), can damage or obstruct the fallopian tubes and increase the risk of infertility [94,95,96,97]. Tubal factors contribute to about 25% of infertility cases, with hydrosalpinx representing the most severe form, often caused by PID [96]. Hydrosalpinx, a pathological condition characterized by the distension of a blocked fallopian tube due to fluid accumulation [98], can reduce implantation and pregnancy rates in assisted reproductive technology (ART) by causing mechanical and chemical disruptions in the tubal microenvironment [96,99]. Tubal function can also be compromised or damaged by salpingitis, a condition often associated with peritubal disease that may result from a ruptured appendix, EM, ectopic pregnancy, or inflammation caused by sexually transmitted infections (STIs) such as *Chlamydia trachomatis* and *Neisseria gonorrhoeae* [100,101].

Endometriosis can cause adhesions between the uterus, ovaries, and fallopian tubes, obstructing the transfer of oocytes to the fallopian tubes and resulting in infertility [94,95,96,97]. In moderate-to-severe endometriosis, fallopian tubes exhibit impaired cilia and weakened contractions [102,103]. Tubal endometriosis, though uncommon, affecting only 4.5 to 6% of women with the condition, leads to several fallopian tube abnormalities, including malformations of the infundibulum (such as phimosis, fimbrial agglutination, and peritubal adhesions), as well as conditions like hydrosalpinx, accessory infundibulum diverticulum, and cornual polyps [104]. Studies have also documented the reduction in sperm motility and impairment in sperm binding to the tubal epithelium in women with endometriosis [105,106,107].

**Table 2:** Impact of medical conditions, infections and pharmacological agents on fallopian tube function.

Compound/Factor	Changes Induced in the Fallopian Tube	Organism	Reference
Endometriosis	Reduced CBF, loss of ciliated cells	Human	Xia et al., 2018 [75]
*Chlamydia trachomatis* and *Neisseria gonorrhoeae* infections	Reduced CBF, loss of ciliated cells	Human	Hafner et al., 2015 [73]; Lenz et al., 2018 [74]
Benign fibroids	Fallopian tube blockage	Human	Olooto et al., 2012 [95]
FGTB	Fallopian tube blockage	Human	Yue et al., 2019 [88]
Antidepressants (escitalopram, paroxetine)	Increased contractions	Human	Milosavljević et al., 2019 [108]
Antiepileptic drugs (carbamazepine, lamotrigine)	Reduced contractions	Human	Jankovic et al., 2006 [109]
Adrenaline	Increased contractions	Human	Senior et al., 1969 [110]; Cibils et al., 1971 [111]
Isoprenaline	Increased relaxation	Human	Senior et al., 1969 [110]
Norepinephrine	Increased proliferation	Human	Dash et al., 2021 [112]
Levonorgestrel, Mifepristone, ulipristal acetate	Reduced CBF	Human, Rat	Zhao et al., 2015 [113]; Christow et al., 2002 [114]
Methotrexate	Tubal disruption, tubal occlusion, chronic damage to the epithelial lining, recurrent ectopic pregnancies, ciliary loss, mitochondrial damage, intracellular edema	Rabbit, Rat	Bayram et al., 2005 [115]; El-Salam et al., 2012 [116]; Cetin et al., 2008 [117]
Cladribine	Apoptosis	Rat	Wawryk-Gawda et al., 2014 [118]
Antiretroviral drugs (ARVs)	Mucosal atrophy	Rat	Awodele et al., 2018 [119]
Verapamil	Spontaneous contractions inhibited	Human	László et al., 1988 [120]
Angiotensin II	Increased CBF	Human	Saridogan et al., 1996 [121]

## 7. Pharmacological Agents

Pharmacological agents are substances that interact with biological systems to alter physiological or biochemical functions for the diagnosis, treatment, or prevention of diseases. The unmonitored overuse or misuse of certain pharmacological agents, or chronic use for treating any medical, metabolic, or pathological conditions, can impair fertility by disrupting fallopian tube function [115,122].

### 7.1. Antidepressants and Antiepileptics

The use of antidepressants among women with depression or anxiety has been shown to reduce fertility and decrease the probability of conception during their reproductive years [123,124]. It has been reported that the SSRI (selective serotonin reuptake inhibitor) class of antidepressants, including escitalopram and paroxetine, significantly stimulate the spontaneous contractions of the ampullary and isthmic segments of the fallopian tubes in women, potentially disrupting their normal functioning [108]. Increased tubal contractions may cause premature sperm transport, while extended isthmic spasms can retain a fertilized oocyte, both contributing to infertility [125]. Jankovic et al. showed that carbamazepine and lamotrigine reduce spontaneous contractions in human ampullar and isthmic segments in a concentration-dependent manner [109].

### 7.2. Sympathomimetic Drugs

Sympathomimetic drugs exert their effects by stimulating adrenergic receptors, which are the targets for the neurotransmitters norepinephrine (noradrenaline) and epinephrine (adrenaline). Studies have demonstrated that stimulation of alpha-receptors by sympathomimetic drugs like adrenaline enhances contractility of the human fallopian tube [110,111]. This increase in contractility is reduced by pre-treatment with alpha-blockers [126]. In contrast, beta-stimulating agents such as isoprenaline induce relaxation of the fallopian tube, whereas norepinephrine can activate both alpha- and beta-adrenergic receptors [110]. Prolonged adrenergic stimulation by norepinephrine has been known to play a role in the metastasis of ovarian carcinoma [112]. Since growing evidence suggests that the fallopian tube epithelium is the origin of most ovarian cancers, Dash et al. conducted a study using a normal immortalized human fallopian tube secretory cell (FTSC) line. The results suggested that prolonged NE exposure could lead to an increase in proliferation and colony-forming ability of FTSCs, potentially contributing to ovarian cancer initiation and progression [112].

### 7.3. Contraceptives

Contraception plays a crucial role in family planning. One of the most commonly used and recommended contraception methods is the oral pill containing levonorgestrel (LNG), a progesterone derivative, which should be taken within 72 h of unprotected sexual intercourse (World Health Organization, 2004) [127]. It has been reported that LNG leads to a significant reduction in the CBF within the fallopian tube [113]. Kopp-Kallner et al. conducted a large, population-based prospective cohort study on women of reproductive age using hormonal contraception, documenting that the risk of ectopic pregnancy was highest among those using LNG hormonal intrauterine devices (IUDs) compared to other hormonal contraceptive methods [128]. Rare pregnancies with progestin-releasing IUDs are more often ectopic, likely due to reduced tubal ciliary activity [129]. Other forms of emergency contraception, such as mifepristone (an anti-progestogen) and ulipristal acetate (UPA) (a selective progesterone receptor modulator), have been reported to inhibit CBF and muscular contractions in the human fallopian tube at pharmacological doses [130]. This inhibition is potentially due to the agonistic effects of mifepristone on tubal progesterone receptors, as it increases progesterone receptor levels in the ampulla and isthmus of the fallopian tube [114].

### 7.4. Anticancer and Antiviral Drugs

Methotrexate (Mtx), a folic acid antagonist and anticancer drug, is also employed for treating other conditions like trophoblastic diseases, tubal ectopic pregnancies, and inducing early abortions. Complications including tubal occlusion (with a reported incidence of 0.9–18.6%) and recurrent ectopic pregnancies (with a reported incidence of 9.1–22.0%) have been associated with the adverse effects of Mtx on the fallopian tube [122]. Calcium channel blockers (e.g., verapamil) inhibit fallopian tube contractions, potentially reducing fertility [120], while angiotensin II increases CBF via its specific receptors [121], which may prematurely transport sperm from the fertilization site [125].

### 7.5. Ovulation Induction Drugs

Ovulation induction is a common practice in women undergoing infertility treatments. Female partners of infertile couples undergo ovulation induction with compounds like clomiphene citrate (CC), letrozole, and gonadotropins for controlled ovarian stimulation as a part of the infertility treatment. Most ovulation induction drugs act on the hypothalamic-pituitary-hormonal axis and can significantly alter the endocrine profile of women undergoing ovarian stimulation, thereby affecting hormone-responsive reproductive organs such as the fallopian tubes. Estrogen levels typically increase during induction with selective estrogen receptor modulators (SERMs) such as CC or tamoxifen (TAM), whereas ovarian stimulation with letrozole, a non-steroidal aromatase inhibitor, reduces serum estrogen levels by blocking aromatase and preventing the conversion of testosterone to estrogen. Table 3 comprises the impact of different ovulation induction drugs on fallopian tube function.

**Table 3:** Impact of ovulation induction drugs on fallopian tube function.

Compound/Factor	Changes Induced in the Fallopian Tube	Organism	Reference
Clomiphene citrate	Tubal hyperplasia and disorganization; Loss of cilia	Human	Cunha et al., 1987 [131]
	Tubal apoptosis; Affecting gamete transport in the fallopian tube; Cilia modulation	Rat	Shao et al., 2009 [132]
Letrozole	Tubal dysplasia; Tubal epithelial multilayering, papillary projections, loss of cilia and nuclear atypia	Rat	Lima et al., 2014 [133]; Abdel-Hamid et al., 2016 [134]
Tamoxifen	Tubal dysplasia; Tubal and ovarian cancer; Tubal hyperplasia and disorganization; Loss of cilia; Affecting gross tubal morphology	Human	Cunha et al., 1987 [131]; Chene et al., 2014 [135]; Pickel et al., 1998 [136]; Tada et al., 2004 [137]
	Tubal hyperplasia	Mouse	Diwan et al., 1997 [138]
	Tubal disruption and dilation; Tubal epithelial multilayering and nuclear atypia	Rat	Lima et al., 2014 [133]; Chamness et al., 1979 [139]
	Reduction in tubal nuclear sites; Affecting egg transport	Rabbit	Gupta et al., 1987 [140]
CC + hCG	Tubal dysplasia; Tubal epithelial multilayering and nuclear atypia	Rat	Lacoste et al., 2013 [141]; Lima et al., 2014 [133]
FSH + hCG	Tubal dysplasia	Rat	Lacoste et al., 2013 [141]
PMSG + hCG	Gynecological cancers; Over-activation of cell cycle proteins in the fallopian tube; Tubal dysplasia and DNA damage	Mouse	King et al., 2011 [142]; Di Nisio et al., 2018 [143]
eCG + hCG	Disruption of tubal embryo transport	Cat	Graham et al., 2000 [144]

#### 7.5.1. Clomiphene Citrate (CC)

CC is the first-line drug used for infertility treatments of women undergoing mild ovulation induction [ovulation induction and timely intercourse; intrauterine insemination (IUI)] and in vitro fertilization (IVF) programs involving mild ovarian stimulation [145,146,147]. CC is a selective estrogen receptor modulator (SERM) and blocks the negative feedback effect of circulating estradiol, which in turn leads to an increased hypothalamic gonadotropin-releasing hormone (GnRH) pulse frequency, subsequently promoting the production of pituitary follicle-stimulating hormone (FSH) and luteinizing hormone (LH), thereby stimulating ovarian follicular growth [148]. Cunha et al. [131] demonstrated that grafted human fetal oviducts in mice treated with CC, TAM, or DES developed epithelial hyperplasia, ciliary loss, and mucosal disruption. Chene et al. observed significant tubal dysplasia in the secretory cells of the fallopian tube in women exposed to CC [135]. Chronic treatment with CC has been documented to be associated with a higher incidence of ectopic pregnancy in humans [149,150,151,152,153,154].

#### 7.5.2. Tamoxifen (TAM)

TAM, structurally similar to CC, is a non-steroidal SERM widely used in breast cancer management [155]. TAM acts as an antiestrogen with a strong affinity for estrogen receptors [156]. It blocks negative feedback of estradiol, increasing GnRH and stimulating FSH and LH production, thus promoting ovarian follicle growth. Chene et al. reported significant tubal dysplasia in the secretory cells of the fallopian tube in TAM-exposed women [135]. The expression of p53 and Ki67, which have been reported to be significantly upregulated in ovarian cancer, exhibited weak expression in ovaries but stronger expression in the fallopian tube of TAM-exposed women. This suggests a key role of the fallopian tube in the pathogenesis of tubal and ovarian cancer.

Further, fallopian tube carcinoma has been reported in postmenopausal women undergoing chronic adjuvant TAM therapy for breast cancer. The fallopian tube epithelium showed bilateral atypical hyperplasia with pleomorphism, cellular crowding, enlarged nuclei and nucleoli, atypia, and nuclear pseudostratification [136,137].

## 8. Conclusions

Women’s reproductive health is increasingly compromised due to exposure to various environmental toxicants, modern lifestyle habits, infections, medical conditions, and pharmacological interventions. These potential threats, whether contributing individually or in combination of multiple risk factors, can significantly disrupt overall reproductive wellbeing. While animal studies provide compelling evidence, human data remains limited, warranting further investigation into the mechanisms underlying these changes. In humans, the most compelling evidence potentially links fallopian tube dysfunction to cigarette smoking, alcohol consumption, bacterial infections (*Chlamydia trachomatis* and *Neisseria gonorrhoeae*), FGTB caused by *Mycobacterium tuberculosis*, conditions such as hydrosalpinx and endometriosis, and interventions including hormonal contraception or ovulation induction with CC or TAM. In light of these challenges, safeguarding women’s reproductive health requires a holistic approach. Minimizing exposure to harmful environmental agents, increasing awareness about toxic agents, promoting healthier lifestyle choices, and exercising caution while using pharmacological agents may help in minimizing the damage to one of the most critical segments of the female reproductive tract.

## Data Availability

Not applicable.

## Abbreviations

The following abbreviations are used in this manuscript:

ART	assisted reproductive technology
ARVs	antiretroviral drugs
BAD	BCL2-associated agonist of cell death
BCL2	B-cell lymphoma 2
BPA	bisphenol A
CBF	ciliary beat frequency
CC	clomiphene citrate
CMV	cytomegalo-virus
DDT	dichlorodiphenyltrichloroethane
DES	diethylstilbestrol
E2	estradiol
eCG	equine chorionic gonadotropin
EDCs	endocrine-disrupting chemicals
FGTB	female genital tuberculosis
FSH	follicle-stimulating hormone
GnRH	gonadotropin-releasing hormone
hCG	human chorionic gonadotropin
HIV	Human Immunodeficiency Virus
HSV	herpes simplex virus
IUI	intrauterine insemination
LH	luteinizing hormone
LNG	levonorgestrel
LET	letrozole
Mtx	methotrexate
MSG	monosodium glutamate
NO	nitric oxide
OVGP1	oviductal glycoprotein 1
P4	progesterone
PID	pelvic inflammatory disease
PMSG	pregnant mare serum gonadotropin
SERM	estrogen receptor modulator
SSRI	selective serotonin reuptake inhibitors
TAM	tamoxifen
TCMP	2,4,6-trichlorophenol
TLRs	Toll-like receptors
TZ	triazophos
UPA	ulipristal acetate
